# Impact of Imaging on Surgical Management of Penetrating Chest Trauma: Experience From a High-Volume Trauma Center in a Resource-Constrained Environment

**DOI:** 10.7759/cureus.77476

**Published:** 2025-01-15

**Authors:** Nosheen Noor, Abdul Baseer

**Affiliations:** 1 Department of Radiology, Medical Teaching Institute, Lady Reading Hospital, Peshawar, PAK; 2 Department of Thoracic Surgery, Medical Teaching Institute, Lady Reading Hospital, Peshawar, PAK

**Keywords:** computed tomography (ct) imaging, penetrating chest trauma, pneumothorax, surgical intervention, trauma management

## Abstract

Background

Penetrating chest trauma is a significant cause of morbidity and mortality, with the need for accurate and timely diagnosis being crucial in determining appropriate management. This study aims to evaluate the role of computed tomography (CT) imaging in the assessment and surgical management of patients with penetrating chest trauma at a high-volume trauma center in a developing country.

Objective

To assess the contribution of CT imaging in the evaluation and surgical management of patients presenting with penetrating chest trauma at a resource-limited trauma center in Pakistan.

Methods

A cross-sectional observational study was conducted in the Thoracic Surgery and Radiology Departments of Lady Reading Hospital, Peshawar, from January 2024 to June 2024. A convenient sampling technique was used to include 139 patients with penetrating chest trauma. Demographic details, trauma complications, fractures, and management strategies were recorded. Thick-slice, non-contrast, chest CT scans were performed and reviewed by a consultant radiologist. Data analysis was carried out using SPSS version 26 (IBM Corp., Armonk, NY).

Results

The study included 139 patients with a mean age of 26.7 ± 8.2 years (range: 8-70 years). The majority were male (126, 90.6%), and the most common mode of injury was firearm trauma (83, 59.7%). The most frequently observed complications were pneumothorax (110, 79.1%), hemothorax (112, 80.6%), and lung injury (88, 63.3%). Fractures were noted in 54 (38.8%) patients, with rib fractures being the most common (31, 22.3%). The most common management approach was tube thoracostomy (116, 83.5%), followed by conservative management (10, 7.2%) and open thoracotomy (6, 4.3%).

Conclusion

CT imaging is an invaluable tool in the assessment of penetrating chest trauma, aiding in the identification of hidden injuries and influencing surgical management decisions. Although thin-slice contrast-enhanced CT is the standard protocol in our resource-constrained setting, non-contrast axial CT scans offer critical diagnostic information and guide timely management. Its use significantly improves patient outcomes, especially in a resource-limited setting.

## Introduction

Trauma and accidents resulting in accidental injuries are the third leading cause of mortality worldwide. They are the primary cause of death among individuals under 45 years of age [1]. Penetrating chest trauma caused by stabbings and gunshots significantly contributes to morbidity and mortality and requires timely, specific management to prevent life-threatening complications [2,3]. In the United States, gunshots and stabbings account for 10% and 9.5% of penetrating chest injuries, respectively. However, this incidence varies globally, reaching up to 95% in war-torn countries [4,5]. Overall, penetrating chest injuries constitute about 10% of trauma admissions, with approximately 10% of these requiring operative interventions [6].

Penetrating chest trauma predominantly affects individuals aged 18 to 65 years, with a higher incidence among males [7]. The severity and prognosis of these injuries depend on factors such as the nature of the penetrating object, its trajectory, and the involvement of vital thoracic structures. Unlike blunt trauma, which causes widespread tissue damage, penetrating injuries result in localized damage to organs such as the lungs, heart, diaphragm, and great vessels [8].

Initial imaging is critical for diagnosing and managing penetrating chest trauma. In resource-constrained environments, anteroposterior (AP) chest radiography is often the first imaging modality employed due to its availability, speed, and utility in identifying life-threatening conditions such as tension pneumothorax, hemothorax, and mediastinal injuries [3].

The American College of Radiology (ACR) recommends chest radiographs as the first-line imaging for penetrating thoracic injuries, regardless of the patient’s hemodynamic stability [9]. A portable chest radiograph performed at the bedside offers rapid visualization of injuries and guides immediate management decisions. While chest radiographs provide essential initial insights, they are primarily used to identify immediate life-threatening conditions such as pneumothorax, hemothorax, and mediastinal widening, which require urgent intervention. However, their limitations must be acknowledged, as they often fail to detect subtle injuries like small pulmonary contusions, diaphragmatic tears, or vascular injuries. Factors such as debris, suboptimal patient positioning, or inadequate inspiration can further compromise image quality. Despite these limitations, chest radiographs are prioritized in trauma settings because they are quick, widely available, and capable of guiding immediate management decisions in resource-constrained environments [10].

CT imaging provides a more sensitive and detailed assessment compared to chest radiographs, allowing for the detection of pulmonary contusions, pneumothorax, hemothorax, diaphragmatic rupture, and vascular trauma that may be overlooked or less distinct on plain radiographs [11]. While chest radiographs can detect many of these findings, CT excels in identifying subtle or complex injuries with greater precision. The limitations on CT use in resource-constrained environments are primarily due to cost and availability, rather than time, as CT scans are typically rapid once the facility and equipment are accessible [8].

This study examines the impact of imaging, specifically non-contrast chest CT, on the surgical management of penetrating chest trauma in a high-volume trauma center operating in a resource-limited setting. It highlights the diagnostic value of non-contrast chest CT in guiding surgical decisions.

## Materials and methods

Study design and setting

This cross-sectional observational study was conducted in the Thoracic Surgery and Radiology Departments of Lady Reading Hospital (LRH), Peshawar, from January 2024 to June 2024. LRH is a high-volume tertiary care center serving a diverse population from urban and rural regions, making it an ideal setting for studying trauma cases.

Sample size and sampling technique

A total of 139 patients with penetrating chest trauma were included in the study by employing a simple convenient sampling technique. The sample size was calculated using the OpenEpi calculator, based on a reported population proportion of 10%, which represents the estimated percentage of penetrating chest trauma cases among all trauma admissions as documented in prior research. This proportion was used to determine a statistically significant sample size for the study.

Inclusion and exclusion criteria

The study included patients admitted to the Thoracic Surgery Department with penetrating chest trauma, defined as injuries caused by objects that pierce the chest wall, such as gunshot wounds (GSWs), stab wounds, or other sharp-force trauma directly penetrating the thoracic cavity. Blunt chest trauma, such as that resulting from vehicle collisions or falls, was excluded to maintain a specific focus on injuries involving direct penetration of the thorax. Patients undergoing chest CT imaging in the Accident and Emergency Department for evaluation of these injuries were also included. Exclusion criteria comprised cases of blunt chest trauma, incomplete medical records, or inadequate imaging data, ensuring the study's focus and data reliability.

Ethical considerations

Ethical approval was obtained from the Institutional Review Board (IRB) of Lady Reading Hospital (Ref No.: 504/LHR/MTI; dated: 02/12/2024). The IRB operates in compliance with the principles outlined in the Declaration of Helsinki and the International Council for Harmonisation of Technical Requirements for Pharmaceuticals for Human Use (ICH) Good Clinical Practice (GCP) guidelines. The confidentiality of all participants was ensured by anonymizing their data and restricting access to it to authorized personnel only, adhering to these internationally recognized ethical standards for biomedical research.

Data collection

Retrospective data of eligible patients were retrieved from the hospital information management system (HIMS). The data collected included patient demographics (age and gender), mode of injury - limited to firearms and stabbing as the mechanisms of penetrating trauma considered in this study, imaging findings, and the surgical management approaches undertaken by the thoracic surgery team. Each patient's medical and imaging records were meticulously reviewed to ensure the accuracy and completeness of the dataset, excluding cases where injuries did not align with the defined inclusion criteria.

Imaging protocols and evaluation

Axial non-contrast chest CT scans were performed in the Emergency Department using an Optima GE 16-slice CT scanner (GE HealthCare, Chicago, IL). The scans were acquired with a slice thickness of 5 to 10 mm, which, while not considered high-resolution imaging, was sufficient for identifying critical thoracic injuries in the trauma setting. These scans were reviewed on the picture archiving and communication system (PACS) by a consultant radiologist, a fellow of the College of Physicians and Surgeons Pakistan (CPSP), ensuring expert analysis. The documented imaging findings included pneumothorax, hemothorax, lung injuries, pneumomediastinum, hemomediastinum, pneumopericardium, diaphragmatic hernia, and fractures of the clavicle, rib, scapula, sternum, vertebrae, or their combinations.

Surgical management

The surgical management approaches recorded were categorized into conservative treatment, chest wall foreign body removal, open thoracotomy, intrapulmonary foreign body removal, diaphragmatic rent repair, and tube thoracostomy.

Statistical analysis

Data analysis was performed using SPSS version 26 (IBM Corp., Armonk, NY). Continuous variables, such as patient age, were expressed as mean ± standard deviation (SD) to represent the variability within the dataset, while categorical variables were summarized as frequencies and percentages to provide an overview of the distribution of discrete data points. A p-value of less than 0.05 was considered statistically significant.

## Results

A total of 139 patients were included, with a mean age of 26.7 years (range: 8-70 years). Most patients were male (126, 90.6%), while females constituted a smaller proportion (13, 9.4%). Firearm injuries were the most frequent mode of trauma (83, 59.7%), followed by stab injuries (56, 40.3%) (Table 1).

**Table 1:** Patient demographics and mode of injury variables. Data are presented as n (%) for categorical variables and mean ± standard deviation (SD) for continuous variables.

Variables	Values (n = 139)	
Gender, n (%)		
Male		126 (90.6)
Female		13 (9.4)
Age (mean ± SD)		26.7 ± 8.2
Mode of injury, n (%)		
Firearm injury		83 (59.7)
Stab injury		56 (40.3)
Total		139 (100)

The most common complications observed were pneumothorax (110, 79.1%), hemothorax (112, 80.6%), and lung injury (88, 63.3%). Pneumomediastinum was present in 16 (11.5%) patients, while hemomediastinum, pneumopericardium, and diaphragmatic hernia occurred in two (1.4%) patients each (Table 2).

**Table 2:** Trauma complications. Data are presented as frequencies, n (%), for all variables.

Common complications	Frequency (%)
Pneumothorax	110 (79.1)
Hemothorax	112 (80.6)
Lung injury	88 (63.3)
Rare complications	
Pneumomediastinum	16 (11.5)
Hemomediastinum	2 (1.4)
Pneumopericardium	2 (1.4)
Diaphragmatic hernia	2 (1.4)
Total complications	139 (100.0)

Fractures were observed in 54 (38.8%) patients, primarily involving ribs (31, 22.3%). Other fractures included rib and scapula (10, 7.2%), vertebral fractures (3, 2.2%), and various combinations with scapula, vertebrae, and sternum (Table 3).

**Table 3:** Types of fractures. Data are presented as frequencies, n (%), for all fracture types.

Type of fracture	Frequency (%)
Rib	31 (22.3)
Rib and scapula	10 (7.2)
Vertebral	3 (2.2)
Clavicle, rib, and scapula	2 (1.4)
Scapula	2 (1.4)
Rib, scapula, and vertebra	1 (0.7)
Rib and sternum	1 (0.7)
Rib and transverse process	1 (0.7)
Rib and vertebral	1 (0.7)
Sternum	1 (0.7)
Sternum and scapula	1 (0.7)
No fractures	85 (61.2)
Total	139 (100.0)

The majority of patients underwent tube thoracostomy (116, 83.5%). Conservative management was applied in 10 (7.2%) cases, while open thoracotomy and foreign body removal were performed less frequently (Table 4).

**Table 4:** Management strategies. Data are presented as frequencies, n (%), for all management approaches.

Management approach	Frequency (%)
Tube thoracostomy	116 (83.5)
Conservative management	10 (7.2)
Open thoracotomy	6 (4.3)
Chest wall foreign body removal	3 (2.2)
Intrapulmonary foreign body removal	3 (2.2)
Diaphragmatic rent repair	1 (0.7)
Total	139 (100.0)

## Discussion

The study provides valuable insights into the patterns of complications observed in penetrating chest trauma cases, particularly focusing on the use of CT imaging for diagnosis and management. The results, which reflect common complications such as pneumothorax, align with the existing literature that frequently highlights pneumothorax as a prevalent issue in thoracic trauma. However, the study does not directly assess or compare the significance of timely diagnosis or management with patient outcomes due to its focus on initial CT findings and management rather than long-term outcomes [12,13].

Pneumothorax was found in 79.1% of the cases, which is consistent with other research emphasizing its common occurrence in penetrating chest trauma. Clinical detection often involves chest radiographs, but these may have limitations, such as false negatives or an inability to detect occult pneumothorax. These limitations further emphasize the importance of CT scans in providing an accurate diagnosis, especially in critically injured patients, where immediate and precise management is crucial for stabilizing the condition. However, as the study focuses solely on penetrating trauma, the comparison between critically injured and other patient groups is not applicable here [14,15].

Hemothorax, the second most common complication (80.6%), is attributed to vascular or lung parenchymal injuries. Firearm injuries, being high-energy trauma, are more likely to result in vascular damage, leading to hemothorax. While chest radiographs are commonly used for detection, a significant accumulation (200-300 mL) is required for visualization. CT scans offer better sensitivity and specificity, making them a valuable diagnostic tool in suspected hemothorax cases [16]. Appropriate management, including tube thoracostomy, remains critical in preventing complications such as fibrothorax or empyema.

Lung injuries, observed in 88 (63.3%) patients, are common in both penetrating and blunt chest trauma. Pulmonary contusions, lacerations, or parenchymal tears often accompany such injuries. These result from direct trauma to the thoracic cavity, leading to alveolar hemorrhage and impaired gas exchange, as previously documented [2].

The management of lung injuries typically involves ensuring adequate oxygenation, addressing complications such as pneumothorax or hemothorax, and surgical repair in severe cases. CT imaging remains the gold standard for detecting and assessing the extent of lung injuries, as radiographs may miss subtle findings [17].

Fractures were observed in 54 (38.8%) patients, with rib fractures being the most common type, affecting 31 (22.3%). Rib fractures are the most frequently encountered skeletal injury in chest trauma due to their anatomical vulnerability. Research shows that rib fractures significantly increase the risk of pneumothorax, hemothorax, and lung contusions, often necessitating tube thoracostomy for associated complications [7,18].

Fractures involving the ribs, scapula, and vertebrae, though observed in a smaller proportion of patients, are typically associated with high-energy trauma, such as motor vehicle accidents or falls. In penetrating chest trauma caused by stab wounds and firearms, these fractures are less commonly encountered compared to blunt trauma. However, when present, vertebral fractures, although rare, warrant careful evaluation for potential spinal instability and neurological deficits. The likelihood of vertebral fractures occurring in penetrating trauma is lower than in blunt trauma, as the mechanism of injury typically affects soft tissues, lung parenchyma, and vasculature rather than the bony structures of the thoracic and spinal regions. Nonetheless, when vertebral fractures do occur in penetrating chest trauma, their clinical significance requires attention to prevent long-term complications [19]. With 61.2% of patients without fractures, this finding underscores the variability in trauma severity, with many cases involving soft tissue injuries or isolated complications like pneumothorax and hemothorax.

Tube thoracostomy was the most common management approach in this study, utilized in 116 (83.5%) patients. This aligns with existing evidence that tube thoracostomy is the gold standard for managing pneumothorax, hemothorax, and other chest trauma complications requiring immediate evacuation of air or blood from the pleural cavity. Studies have demonstrated its efficacy in restoring lung expansion and preventing tension pneumothorax or further pulmonary complications [20].

Conservative management was employed in 10 (7.2%) cases. This strategy is typically reserved for patients with small pneumothorax, hemothorax, or other minor chest trauma. Published research indicates that conservative management can be effective in carefully selected cases, reducing the need for invasive interventions [21].

Open thoracotomy and foreign body removal were performed in six (4.3%) patients each. These procedures are generally indicated for severe injuries such as massive hemothorax, persistent bleeding, or retained foreign bodies that cannot be managed without the use of invasive techniques. Previous studies highlight that quick surgical involvement in such cases improves survival rates and minimizes complications [12].

The observed rates of pneumothorax and hemothorax are higher than those reported for blunt chest trauma, reflecting the distinct mechanisms and severity of penetrating injuries. Literature highlights that penetrating trauma often causes direct and extensive damage to thoracic structures, resulting in higher complication rates compared to blunt trauma [16,22]. The study's demographic distribution, with a predominance of young male patients, mirrors global trends in trauma epidemiology, as males are more frequently exposed to violence and occupational hazards.

While thin-slice, multiplanar CT with contrast is generally considered the standard protocol for imaging penetrating trauma [23], in a resource-constrained environment, non-contrast axial CT scans with slice thicknesses of 5-10 mm, as utilized in this study, can still provide valuable diagnostic insights. Although these non-contrast scans are not high-resolution "thin section" images, they are a practical and effective alternative when advanced imaging resources are limited. In our setting, these CT scans offered essential information that guided clinical decision-making, demonstrating their utility in situations where higher-resolution imaging might not be feasible. Given the high volume of trauma patients in this largest tertiary care public hospital in the region and the provision of free emergency services, the hospital cannot afford intravenous contrast for all such patients to follow international protocols. Non-contrast CT scans are proven effective in our setting, offering critical diagnostic information to guide timely and appropriate management decisions.

This study is limited by its retrospective nature and reliance on single-center data, which may affect the generalizability of findings. Future studies should explore long-term outcomes, such as the impact of initial injury management on recovery and complications over time. Additionally, the role of advanced imaging techniques, including point-of-care ultrasound, in the early detection of complications, has been well-documented in the literature and warrants further exploration. This could help improve the timely identification of injuries in environments where CT scans may not be immediately available.

## Conclusions

This study highlights the critical role of early diagnosis and intervention in managing penetrating chest trauma, with radiological imaging, particularly chest CT scans, proving indispensable in identifying injuries and guiding clinical decisions. Tube thoracostomy emerged as the cornerstone treatment, effectively managing most complications, while surgical approaches like thoracotomy were reserved for severe cases.

The value of non-contrast axial CT imaging in resource-constrained settings was a key finding, demonstrating its practical utility in providing essential diagnostic insights and facilitating timely management. These results underscore the need for tailored therapeutic strategies and a multidisciplinary approach to optimize outcomes in patients with penetrating chest injuries.
